# Ca^2+ ^regulation in the absence of the *iplA *gene product in *Dictyostelium discoideum*

**DOI:** 10.1186/1471-2121-6-13

**Published:** 2005-03-11

**Authors:** Ralph H Schaloske, Daniel F Lusche, Karen Bezares-Roder, Kathrin Happle, Dieter Malchow, Christina Schlatterer

**Affiliations:** 1 Department of Chemistry and Biochemistry, University of California at San Diego, 9500 Gilman Drive, La Jolla, CA 92093-0601, USA; 2 Faculty for Biology, University of Konstanz, 78457 Konstanz, Germany

## Abstract

**Background:**

Stimulation of *Dictyostelium discoideum *with cAMP evokes an elevation of the cytosolic free Ca^2+ ^concentration ([Ca^2+^]_i_). The [Ca^2+^]_i_-change is composed of liberation of stored Ca^2+ ^and extracellular Ca^2+^-entry. The significance of the [Ca^2+^]_i_-transient for chemotaxis is under debate. Abolition of chemotactic orientation and migration by Ca^2+^-buffers in the cytosol indicates that a [Ca^2+^]_i_-increase is required for chemotaxis. Yet, the *iplA*^- ^mutant disrupted in a gene bearing similarity to IP_3_-receptors of higher eukaryotes aggregates despite the absence of a cAMP-induced [Ca^2+^]_i_-transient which favours the view that [Ca^2+^]_i_-changes are insignificant for chemotaxis.

**Results:**

We investigated Ca^2+^-fluxes and the effect of their disturbance on chemotaxis and development of *iplA*^- ^cells. Differentiation was altered as compared to wild type amoebae and sensitive towards manipulation of the level of stored Ca^2+^. Chemotaxis was impaired when [Ca^2+^]_i_-transients were suppressed by the presence of a Ca^2+^-chelator in the cytosol of the cells. Analysis of ion fluxes revealed that capacitative Ca^2+^-entry was fully operative in the mutant. In suspensions of intact and permeabilized cells cAMP elicited extracellular Ca^2+^-influx and liberation of stored Ca^2+^, respectively, yet to a lesser extent than in wild type. In suspensions of partially purified storage vesicles ATP-induced Ca^2+^-uptake and Ca^2+^-release activated by fatty acids or Ca^2+^-ATPase inhibitors were similar to wild type. Mn^2+^-quenching of fura2 fluorescence allows to study Ca^2+^-influx indirectly and revealed that the responsiveness of mutant cells was shifted to higher concentrations: roughly 100 times more Mn^2+ ^was necessary to observe agonist-induced Mn^2+^-influx. cAMP evoked a [Ca^2+^]_i_-elevation when stores were strongly loaded with Ca^2+^, again with a similar shift in sensitivity in the mutant. In addition, basal [Ca^2+^]_i _was significantly lower in *iplA*^- ^than in wild type amoebae.

**Conclusion:**

These results support the view that [Ca^2+^]_i_-transients are essential for chemotaxis and differentiation. Moreover, capacitative and agonist-activated ion fluxes are regulated by separate pathways that are mediated either by two types of channels in the plasma membrane or by distinct mechanisms coupling Ca^2+^-release from stores to Ca^2+^-entry in *Dictyostelium*. The *iplA*^- ^strain retains the capacitative Ca^2+^-entry pathway and an impaired agonist-activated pathway that operates with reduced efficiency or at higher ionic pressure.

## Background

Aggregation of *Dictyostelium discoideum *proceeds by an oriented migration of the amoebae towards a source of the attractant cAMP which is synthesized and released periodically by cells in the center of the aggregate. Stimulation with cAMP activates liberation of stored Ca^2+ ^and extracellular Ca^2+^-entry [1] leading to a [Ca^2+^]_i_-transient [2-4]. Chemotaxis proceeds in the presence of extracellular EGTA but not in the presence of intracellular Ca^2+ ^buffers, so a [Ca^2+^]_i_-elevation is necessary and release of stored Ca^2+ ^is sufficient for oriented migration [5]. On the other hand, the view that a [Ca^2+^]_i_-increase is essential for chemotaxis was called into question by analysis of a cell line where the *iplA *gene was disrupted by homologous recombination [6]. The *iplA *gene is the only gene known in the *Dicyostelium *genome so far that shares homology with IP_3_-receptors of higher eukaryotes. However, whether its protein product indeed constitutes a functional IP_3_-receptor and its cellular localization are not known. The *iplA*^- ^mutant was found to aggregate and to form fruiting bodies although neither cAMP-activated ^45^Ca^2+^-entry nor a [Ca^2+^]_i_-elevation were detected [6]. From these results the authors concluded that the *iplA *gene product is central to the regulation of [Ca^2+^]_i _and that its presence and thus the presence of an agonist-activated [Ca^2+^]_i_-increase is not necessary for proper chemotaxis and development. However, agents that interfere with IP_3_-receptor mediated signaling such as XestosponginC [7] were found to influence not only cAMP-induced Ca^2+^-fluxes but also the chemotactic response and aggregation of *Dictyostelium *[8]. In this study we aimed to clarify these conflicting findings and analyzed both, capacitative and chemoattractant-induced Ca^2+^-fluxes and the effect of their disturbance on chemotaxis and differentiation of the *iplA*^- ^mutant. Mn^2+^-influx was used to determine the rates of ion fluxes into cells with filled and emptied stores and related to Ca^2+^-electrode recordings in cell suspensions. We found that ion fluxes, chemotaxis and differentiation were sensitive towards alteration of the Ca^2+^-homeostasis. Capacitative Ca^2+^-entry was normal in the mutant and upon stimulation with agonist Ca^2+^- and Mn^2+^-fluxes occurred, yet to a considerably reduced extent. Spontaneous motility and chemotactic performance of mutant amoebae was strongly impaired by the intracellular presence of a Ca^2+^-chelator.

## Results

### Extracellular [Ca^2+^] affects development and chemotaxis of wild type and *iplA*^-^

**Figure 1:**
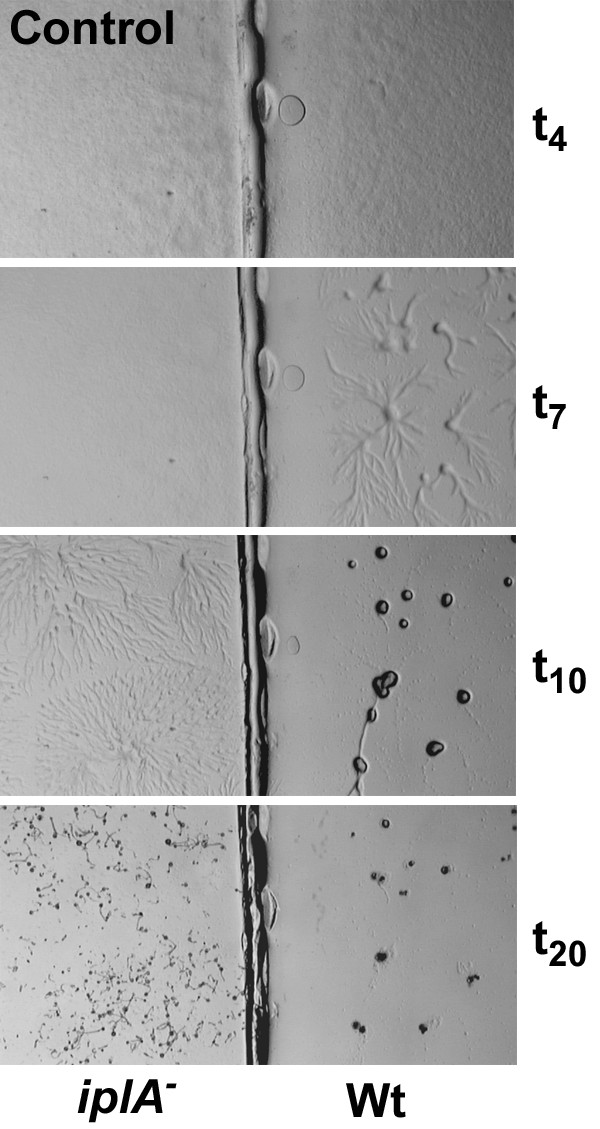
*iplA*^- ^cells have an altered pattern of development. Differentiation of the mutant and the wild type strain was assayed in parallel on agar plates. Cells at different time points of development on H5-agar are shown. Wild type amoebae aggregated at t_7_, whereas aggregation of the mutant strain was delayed and aggregation strands broke (t_10_); therefore, smaller fruiting bodies were formed as compared to the wild type. The full width of the image corresponds to 12.5 mm.

**Figure 2:**
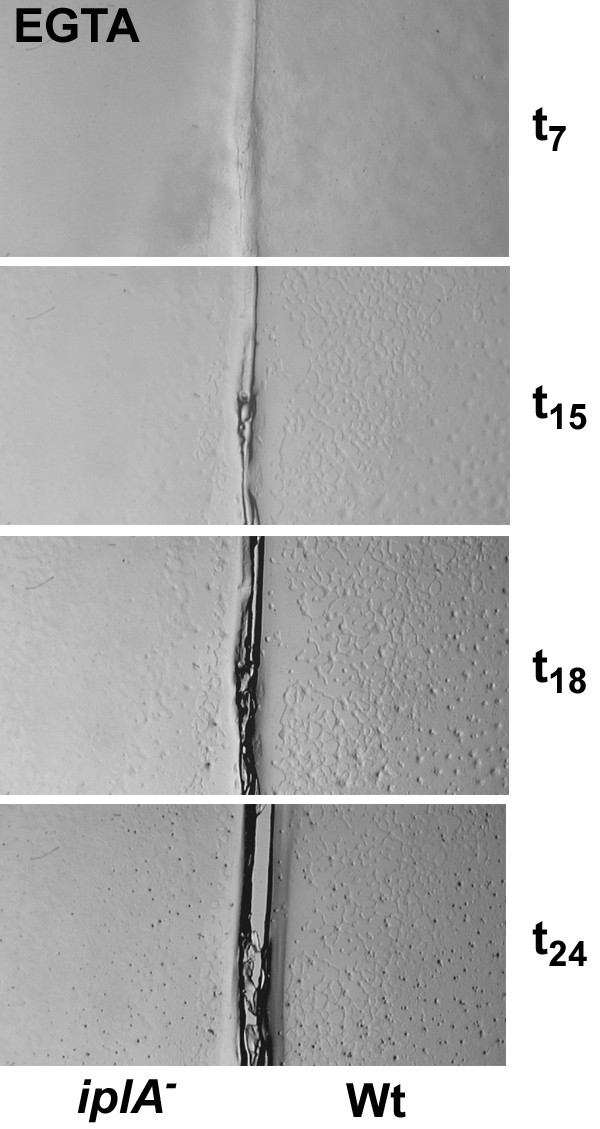
Development of *iplA*^- ^cells is impaired by depletion of internal Ca^2+^-stores due to EGTA-treatment. Differentiation of the wild type and the mutant on plates containing 20 mM EGTA is shown. Aggregation was delayed in both strains till t_15 _and t_18 _in wild type and *iplA*^- ^cells, respectively. The size of the aggregates and the fruiting bodies were much smaller than under control conditions. The full width of the image corresponds to 12.5 mm.

**Figure 3:**
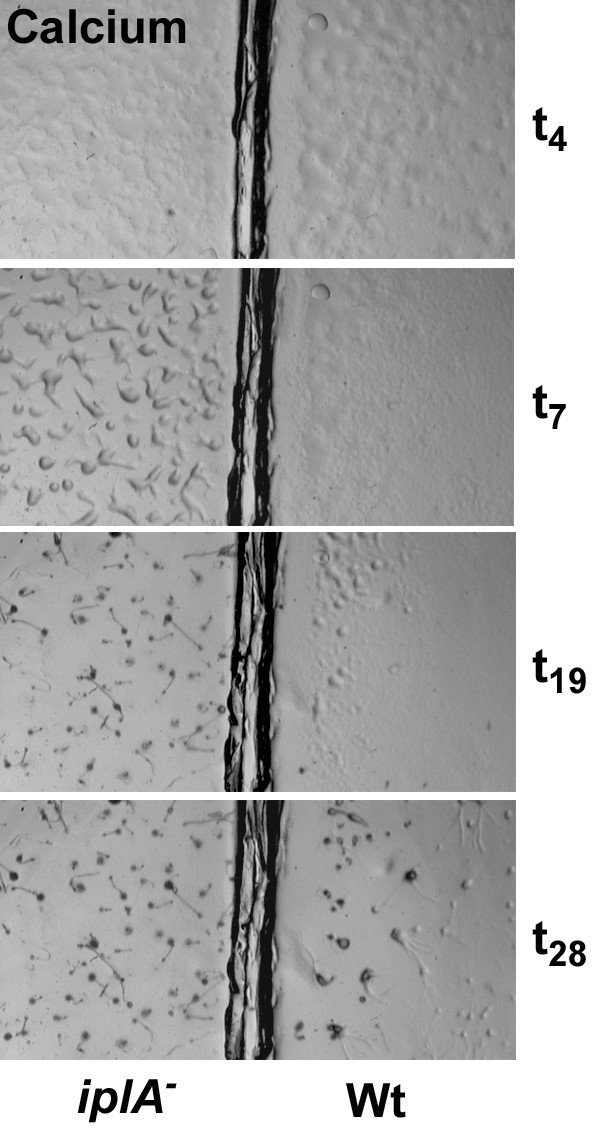
In the presence of external Ca^2 ^aggregation is accelerated in *iplA*^- ^cells. Differentiation of the mutant and the wild type strain was assayed in parallel on agar plates supplemented with 20 mM CaCl_2_. Aggregate formation occurred earlier in *iplA*^- ^cells (at t_7_) than in wild type (starting at t_19_) in the presence of Ca^2+^. The full width of the image corresponds to 12.5 mm.

As *iplA*^- ^cells formed fruiting bodies, albeit somewhat smaller in size, it was concluded that chemotactic aggregation and differentiation was normal [6]. We analyzed development of the mutant in parallel with wild type at various conditions. When cells differentiated on H5-agar plates (control situation) we consistently found a delay in the onset of aggregation by 1–2 h in the mutant; the smaller size of fruiting bodies was due to breaking of aggregation strands yielding smaller mounds (Fig. 1). Next we asked whether the absence or presence of Ca^2+ ^affects development. Differentiation on EGTA-containing agar plates and thus the steady reduction of internal Ca^2+^-levels dose dependently resulted in a delay of aggregation and a decrease in the size of aggregates and fruiting bodies in both strains. Doses of 5–10 mM EGTA in the agar did not significantly alter the time point of aggregation which is in accordance with previous data [9] showing requirement of additional multiple washing of amoebae with EGTA in order to affect aggregation. At concentrations of 15–20 mM EGTA however, aggregation occurred at later time points (Fig. 2), on average at 13 ± 3 h in wild type and at 19 ± 2 h in *iplA*^- ^cells (mean ± s.e.m. from 5 experiments); despite the daily variations in aggregation timing the mutant strain was delayed as compared to the wild type in each of the experiments performed. On the other hand, the presence of Ca^2+ ^in the agar and therefore the continuous loading of cells with Ca^2+ ^[10] resulted in stronger impairment of aggregation in the wild type. The delayed aggregation of wild type cells in the presence of Ca^2+ ^was not due to inhibition of chemotaxis (see below). Now the formation of aggregates was observed consistently at earlier time points in the *iplA*^- ^strain than in wild type (Fig. 3; on average 7 ± 0.5 h vs. 15 ± 5 h until aggregate formation in *iplA*^- ^and wild type amoebae, respectively, at 20 mM CaCl_2 _in 6 independent experiments); indeed, under this condition differentiation of *iplA*^- ^cells resembled that of wild type observed in the control situation.

**Figure 4:**
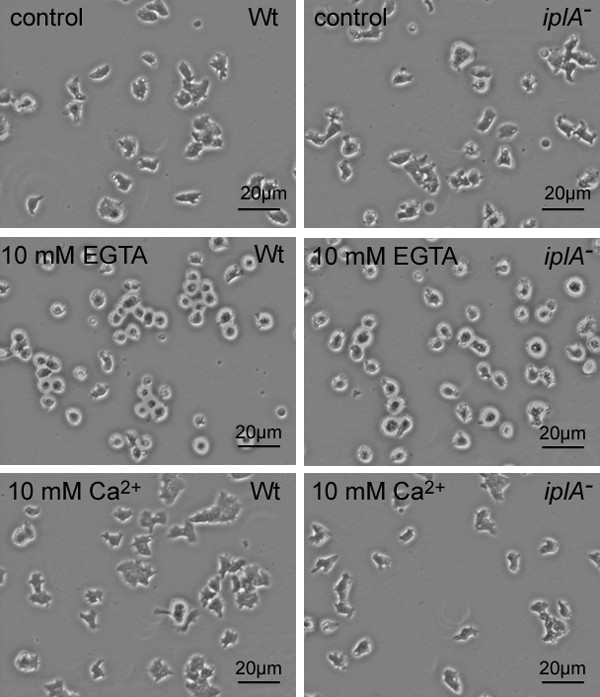
General morphology of wild type and *iplA*^- ^cells under control conditions (A, B), in the presence of 10 mM EGTA for 60 min (C, D) or in the presence of 10 mM CaCl_2 _for 80 min (E, F). In H5-buffer or in the presence of 10 mM CaCl_2 _the morphology was not significantly different between wild type and mutant amoebae. However, in the presence of EGTA the cells of both strains were rounded. Photographs were taken at t_5_. Basal motility under these conditions can be viewed in the accompanying movies.

**Figure 5:**
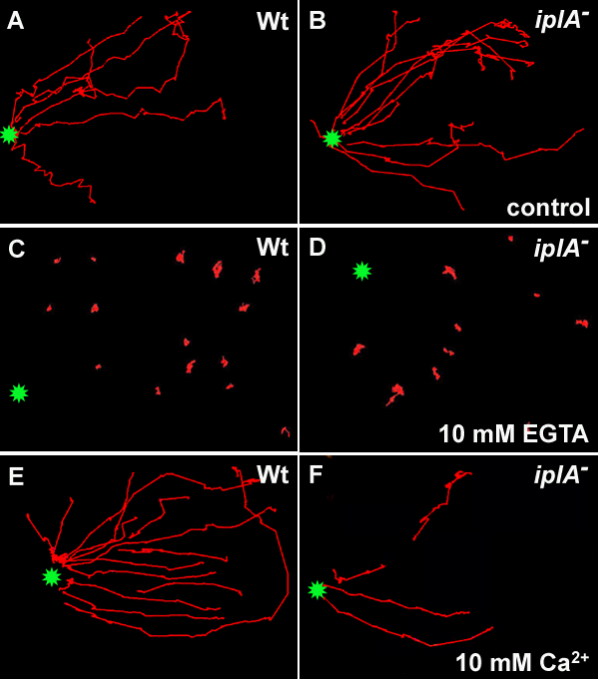
Chemotaxis of wild type and mutant amoebae at different experimental conditions. The tracks of individual cells (in red) migrating during chemotactic stimulation (position of the tip of the cAMP-filled capillary: green star) are shown. In H5-buffer (A, B) both cell types migrated in an oriented manner towards the capillary tip, albeit not always in a straight line. After preincubation with 10 mM EGTA for 60 min and in its continued presence during the chemotaxis assay (C, D) the cells remained stationary with random pseudopod extension. Preincubation of amoebae with 10 mM CaCl_2 _(E, F) did not impair chemotaxis; rather, the cells of both strains migrated towards the capillary tip. Chemotaxis experiments were done at t_6_.

Then we analyzed the effect of treatment with either EGTA or Ca^2+ ^on basal cell motility. We found that under control conditions the general morphology of the cells as well as extension of pseudopods was practically identical in both strains (Fig. 4 A, B, [see Additional file 1]). Preincubation with 10 mM EGTA for 60 min led to strong rounding of wild type and mutant amoebae (Fig. 4 C, D) with reduced extension of small pseudopods [see Additional file 2]. By contrast, pretreatment with 10 mM CaCl_2 _did not affect the morphology (Fig. 4 E, F) or the extension of pseudopods [see Additional file 3] in both strains. Next we tested chemotaxis of amoebae towards a cAMP-filled glass capillary. Under control conditions cells of both strains oriented and migrated towards the tip of the capillary (Fig. 5 A, B); the average chemotactic speed was not different between wild type and the mutant strain (10.7 ± 2.1 vs. 11.0 ± 0.7 μm/min; mean ± s.e.m. of 20 wild type and 43 mutant amoebae analyzed in 3 and 4 independent experiments, respectively). Incubation in 10 mM EGTA for 60 min abolished chemotaxis in most of the wild type and the *iplA*^- ^amoebae: small pseudopods were extended randomly and the cells did not approach the capillary tip (Fig. 5 C, D). Only rarely, cells of both strains exhibited an oriented but highly reduced migration towards the cAMP source (3% and 5% of 33 wild type and 19 mutant cells analyzed in 3 independent experiments, respectively). Thus the loss of Ca^2+ ^from stores impairs both, orientation and migration also in the absence of the *iplA *gene product. By contrast, when amoebae were incubated in 10 mM CaCl_2 _for 60 min to load stores, they oriented and migrated towards the cAMP capillary (Fig. 5 E, F). The chemotactic speed of wild type cells (9.9 ± 1.2 μm/min; mean ± s.e.m. of 17 cells tested in 3 independent experiments) was comparable to that under control conditions whereas mutant amoebae chemotaxed significantly faster (13.6 ± 1.7 μm/min; mean ± s.e.m. of 15 mutant amoebae analyzed in 3 independent experiments) than wild type cells in the presence of 10 mM CaCl_2 _(Mann-Whitney rank sum test, p = 0.041).

### Buffering of intracellular [Ca^2+^] impairs chemotaxis

**Figure 6:**
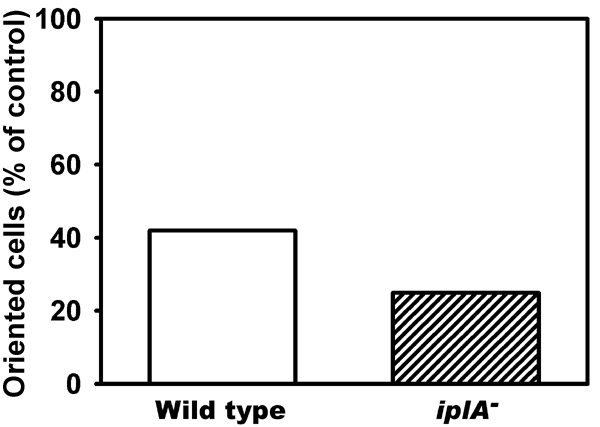
Chemotaxis of *iplA*^- ^cells is impaired in the intracellular presence of a Ca^2+^-buffer. Wild type and mutant amoebae were loaded with Fura2-dextran and their ability to protrude pseudopods towards a cAMP-filled glass capillary was compared to that of untreated cells. In both strains the presence of the chelator in the cytosol led to a decrease in the fraction of cells extending pseudopods and migrating towards the cAMP source.

The observation that aggregation occurred in the mutant cell line although a cAMP-activated increase in [Ca^2+^]_i _was not detectable resulted in the conclusion that [Ca^2+^]_i_-changes were not necessary to accomplish chemotaxis [6]. We used the mobile buffer approach originally described by Speksnijder et al. [11] which allows to analyze the requirement of a [Ca^2+^]_i_-gradient for a given response. If in *Dictyostelium *a [Ca^2+^]_i_-increase was necessary for chemotaxis, the presence of a Ca^2+^-chelator in the cytosol should impair orientation and/or migration. In a previous study, we had introduced the Ca^2+^-chelator BAPTA and its derivatives into the cytosol of wild type amoebae which indeed had inhibited chemotactic migration and reduced chemotactic orientation [5]. Here we used the Ca^2+^-indicator Fura2-dextran to clamp [Ca^2+^]_i _and loaded the indicator into wild type and mutant cells in the absence of external CaCl_2_. The treatment affected chemotactic performance of wild type as well as *iplA*^- ^amoebae. Lack of extracellular CaCl_2 _during the loading process induced strong rounding of the amoebae and loss of migration. Figure 6 shows that the capacity to orient chemotactically and to extend pseudopods towards the capillary tip was reduced by 58% in wild type (93 cells tested in 4 independent experiments). Inhibition was also evident in *iplA*^- ^cells (Fig. 6) showing sensitivity of the mutant towards buffering of intracellular Ca^2+^-levels and eradication of [Ca^2+^]_i_-changes: the fraction of pseudopods extended in direction of the cAMP-source was reduced by 75% (74 cells tested in 3 independent experiments). These results show that not only in wild type but also in the *iplA*^- ^cell line the ability to orient and to migrate in fact depends on an agonist-activated [Ca^2+^]_i_-elevation.

### Analysis of Ca^2+^-fluxes

**Figure 7:**
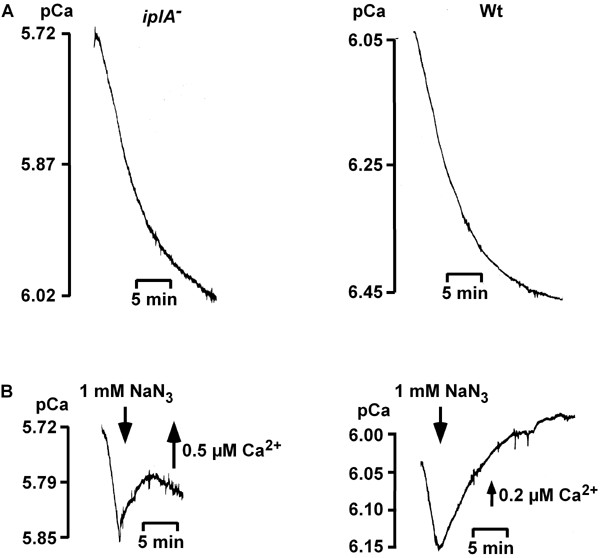
Recordings of Ca^2+^-fluxes in *iplA*^- ^and wild type amoebae. [Ca^2+^]_e _was measured in cell suspensions with a Ca^2+^-sensitive electrode. (A) Treatment of amoebae with 5 mM EGTA for 30 min activated capacitative Ca^2+^-influx (one out of 12/6 determinations in 4/3 independent experiments is shown for *iplA*^- ^and wild type, respectively). (B) Capacitative influx was blocked by the addition of 1 mM NaN_3 _(one out of 5/4 determinations in 3/3 independent experiments). Measurements were done at t_3_.

Our findings that differentiation was sensitive towards depletion of Ca^2+ ^or loading of the cells with Ca^2+ ^and that chemotaxis was blocked by intracellular Ca^2+^-buffers led us to investigate Ca^2+^-fluxes in the mutant cell line. We used a Ca^2+^-sensitive electrode in cell suspensions to measure Ca^2+^-fluxes, an approach different from that of Traynor et al. [6] who had studied ion fluxes by ^45^Ca^2+^-measurements. First we tested whether the coupling of stores to the plasma membrane, i.e. capacitative Ca^2+^-fluxes without prior stimulation with agonists were altered. In *Dictyostelium *induction of capacitative Ca^2+^-influx requires active intracellular Ca^2+^-pumps. Their inhibition by either thapsigargin or 2,5-di-(t-butyl)-1,4-hydroquinone (BHQ) does not evoke influx; rather, stores have to be emptied by treatment with EGTA [12]. Capacitative Ca^2+^-fluxes were studied early during differentiation (t_2_–t_4_). At this time Ca^2+^-influx and Ca^2+^-efflux are at an equilibrium which held true for both, wild type and mutant cells (not shown); in suspensions of cells at later stages of development influx strongly exceeds efflux [1]. In *iplA*^- ^and in wild type cells emptying of storage compartments via preincubation of amoebae with EGTA induced capacitative Ca^2+^-entry (Fig. 7 A) which was blocked by addition of 1 mM NaN_3 _(Fig. 7 B). The characteristics of influx were comparable in wild type and mutant cells. These data show that capacitative Ca^2+^-influx does not depend on the product of the *iplA *gene.

**Figure 8:**
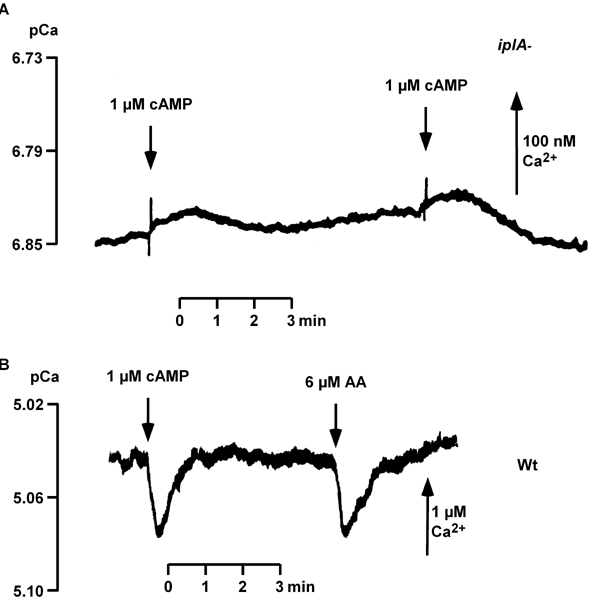
Agonist-activated Ca^2+^-fluxes in suspensions of *iplA*^- ^and wild type amoebae. cAMP elicited reversible Ca^2+^-influx in the mutant (A) and in wild type ((B) and see [14]); measurements were done at t_7_–t_7.5_. Note the different doses of CaCl_2 _added for calibration. The time points of cAMP-addition (1 μM) and of AA-addition (6 μM) in the wild type are indicated by arrows.

**Figure 9:**
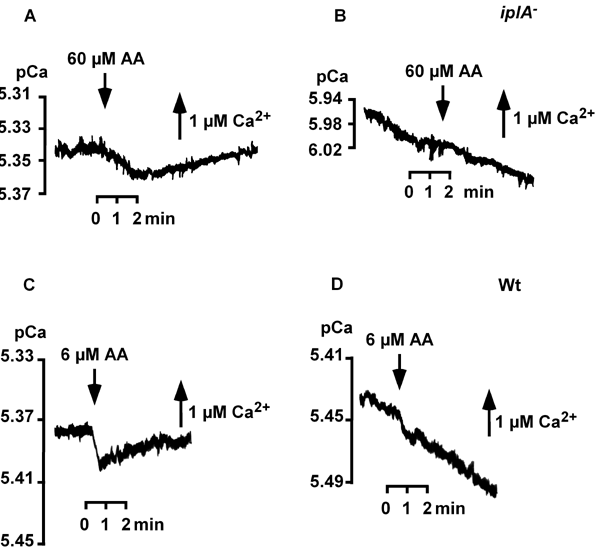
Fatty acids activate Ca^2+^-fluxes in *iplA*^- ^amoebae. (A) 60 μM AA evoked a transient decrease in [Ca^2+^]_e _representing Ca^2+^-influx; measurement was done at t_6 _(B) After preincubating amoebae with the SERCA-type Ca^2+^-ATPase blocker BHQ (100 μM) for 20 min to inhibit uptake of Ca^2+ ^into internal storage compartments, the AA-activated response was absent; measurement was done at t_7.5_. Results of measurements with wild type (C, D) stimulated with 6 μM AA are shown for comparison.

On the other hand, agonist-activated ^45^Ca^2+^-entry had been reported to be absent in the mutant strain; in their study, Traynor et al. had stimulated cells with cAMP in the presence of 0.1 mM CaCl_2 _[6]. The use of a Ca^2+^-sensitive electrode allows to measure much lower levels of extracellular Ca^2+ ^to analyze Ca^2+^-fluxes, in the range of approximately 1 μM Ca^2+^. Indeed, we found that under this condition reversible Ca^2+^-entry occurred after addition of 1 μM cAMP (Fig. 8 A) that amounted to 10.2 ± 4.3 pmol Ca^2+^/10^7 ^cells (mean ± s.d. from 9 experiments). The level of influx represented roughly 5% of wild type influx (Fig. 8 B and [13,14]). Ca^2+^-influx was delayed in the mutant and the time to reach the maximum was longer than in wild type cells [13,14]. In addition, challenge with arachidonic acid (AA) induced influx (Fig. 9 A). Again, the mutant was less sensitive and higher concentrations were required than those reported to evoke Ca^2+^-entry in wild type cells (Fig. 8 B, 9 C and [13]). Neither 10 nor 20 μM AA were effective; in the wild type 10 μM AA activates influx of 190 ± 58 pmol Ca^2+^/10^7 ^cells [14]. At 60 μM AA entry occurred in the mutant strain which amounted to an average of 107 ± 21 pmol Ca^2+^/10^7 ^cells (mean ± s.d. from 7 experiments). Preincubation of cells with the SERCA-type Ca^2+^-ATPase inhibitor BHQ reduced AA-induced influx by 82 % (Fig. 9 B) to an average of 21 ± 18 pmol Ca^2+^/10^7 ^cells (mean ± s.d. from 3 experiments). These data show that Ca^2+^-fluxes across the plasma membrane do occur in *iplA*^- ^cells as well but at a reduced level.

**Figure 10:**
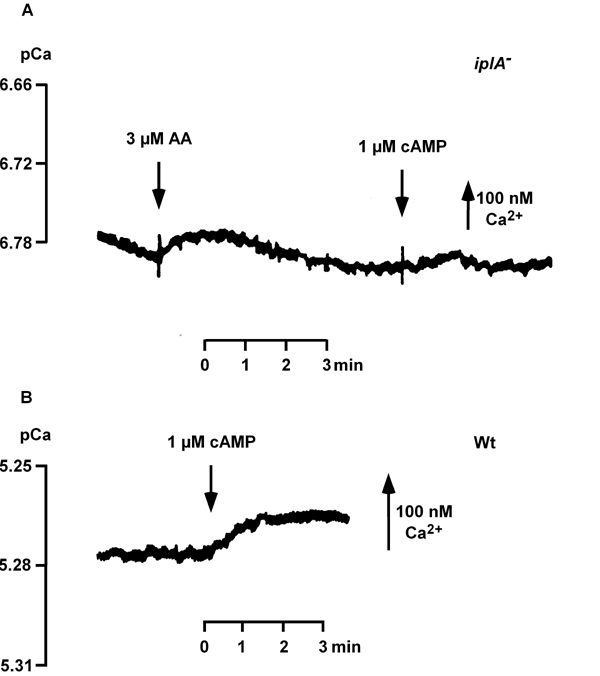
cAMP and arachidonic acid elicit Ca^2+^-release from internal stores. (A) [Ca^2+^]_e _was recorded at t_7 _in *iplA*^- ^cells with permeabilized plasma membranes. Amoebae were challenged with 1 μM cAMP and 3 μM AA, respectively. (B) The response of permeabilized wild type stimulated with 1 μM cAMP at t_6 _is shown for comparison.

Next we tested whether the mutant strain was able to release stored Ca^2+ ^when stimulated with cAMP or AA. Fluxes were measured in suspensions of cells with permeabilized plasma membranes; any change in [Ca^2+^]_e _thus reflects efflux of Ca^2+ ^from storage compartments. Both, cAMP and arachidonic acid activated release of stored Ca^2+ ^(Fig. 10 A). On average, addition of 1 μM cAMP released 7.3 ± 3.4 pmol Ca^2+^/10^7 ^cells and 16.3 ± 7.2 pmol Ca^2+^/10^7 ^cells were liberated after stimulation with 3 μM AA (mean ± s.d. from 10 and 3 experiments, respectively). The amount of Ca^2+^-efflux from stores after cAMP stimulation was 61% of that found in wild type cells (Fig. 10 B and [15]) whereas release upon AA-challenge was in the range of 5–10% of wild type (see Fig. 2 in [14]). In addition to the Ca^2+^-electrode recordings, we studied Ca^2+^-fluxes in suspensions of partially purified storage compartments fluorimetrically. ATP induced Ca^2+^-sequestration (Table 1) was of similar magnitude and rate as in wild type stores. This result indicates that the decreased release of Ca^2+ ^from the stores measured with the Ca^2+^-electrode is not due to a lack of storage capacity. Moreover, the addition of AA evoked release of Ca^2+ ^from stores as did inhibition of Ca^2+^-pump(s) by thapsigargin. XestosponginC that inhibits Ca^2+^-uptake and activates Ca^2+^-release in wild type [8] and the ionophore ionomycin also resulted in substantial Ca^2+ ^release in the mutant cell line (Table 1). All values were in the same range as those for wild type.

**Table 1:** Determination of Ca^2+^-fluxes in partially purified storage compartments of the *iplA*^- ^mutant and of wild type. Ca^2+^-sequestering vesicles were prepared as outlined in Methods. Measurements were performed with the pellet and supernatant fraction. ATP-induced uptake and release activated by different agents is given as nmol Ca^2+^-uptake/min and mg of protein and pmol Ca^2+^-release/tube, respectively (mean ± s.d.). In release experiments 60–75 μl of pellet and 120–140 μl of supernatant fraction were used per tube. Numbers in brackets give number of experiments; n.d.: not determined.

	*iplA*^-^		Wt	
Stimulation	Fraction		Fraction	
	Pellet	Supernatant	Pellet	Supernatant
Uptake (nmol/min*mg)				
1 mM ATP	1.96 ± 0.55 (4)	0.28 ± 0.15 (4)	1.87 ± 0.74 (3)	0.38 ± 0.16 (3)
				
Release (pmol/tube)				
10 μM AA	360 ± 227 (8)	761 ± 218 (6)	396 ± 122 (6)	961 ± 374 (2)
40 μM Thapsigargin	570 ± 250 (6)	170 ± 73 (6)	582 ± 123 (5)	265 ± 135 (2)
6 μM XestosponginC	201 ± 55 (4)	n.d.	276 ± 46 (3)	n.d.
2 μM Ionomycin	447 ± 147 (5)	147 ± 41 (4)	580± 173 (3)	193 ± 76 (2)

### Mn^2+^-quenching experiments

**Figure 11:**
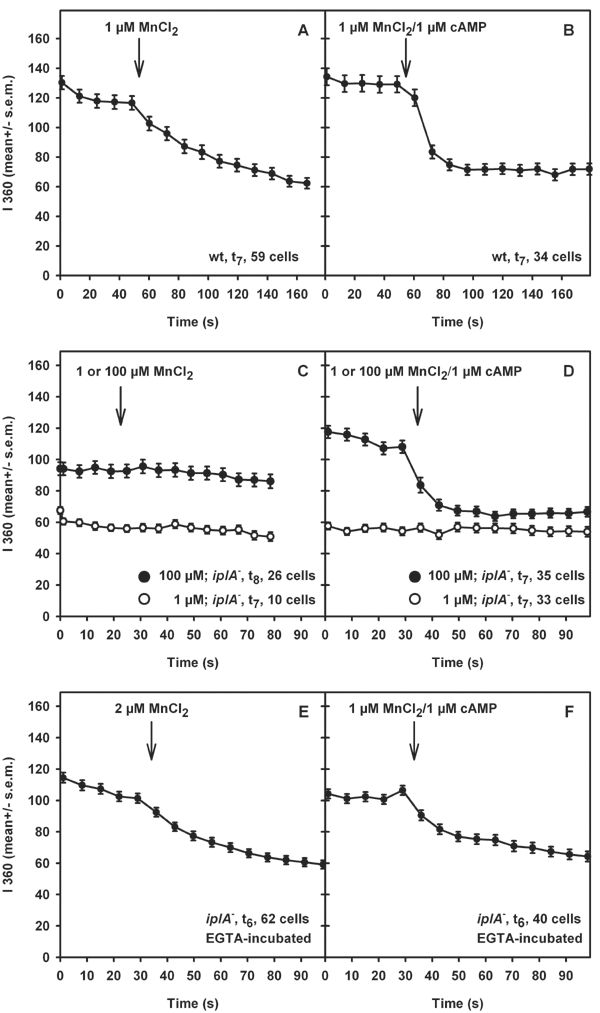
Basal and cAMP-induced Mn^2+^-influx. Influx was assayed by quenching of Fura2-dextran fluorescence. (A, B) The response of wild type amoebae is shown for comparison; 1 μM Mn^2+ ^± 1 μM cAMP was added. *iplA*^- ^cells in nominally Ca^2+^-free buffer were challenged with 100 μM Mn^2+ ^± 1 μM cAMP at t_7 _(closed symbols); when 1 μM Mn^2+ ^was added (open symbols) no influx was detected (C, D). After preincubation with EGTA influx was observed at 1–2 μM Mn^2+ ^± 1 μM cAMP (E, F). Fluorescence intensity at 360 nm excitation is shown as mean ± s.e.m

The [Ca^2+^]_e_-recordings in suspensions of cells as described above detect the sum of Ca^2+^-influx and efflux. Therefore, a complementary approach which monitors influx only was pursued, by using the Mn^2+^-quenching technique in single intact amoebae. This method is based on the fact that many Ca^2+^-channels are permeable to Mn^2+ ^[16] and that the Ca^2+^-indicator Fura2 binds Mn^2+ ^with high affinity. Fluorescence of the indicator is quenched upon binding [17]. We compared quenching of Fura2-dextran fluorescence activated by addition of Mn^2+ ^alone or in combination with 1 μM cAMP in wild type and mutant cells. Higher concentrations of MnCl_2 _were required to quench Fura2-dextran fluorescence in *iplA*^- ^amoebae (Fig. 11, Table 2). Reduction of fluorescence occurred at 1 μM Mn^2+ ^and 1 μM Mn^2+^/cAMP in wild type (Fig. 11 A, B; see also [12]) but not in the mutant (Fig. 11 C, D) where addition of 100 μM Mn^2+^/cAMP was necessary; 100 μM Mn^2+ ^alone was not effective (Fig. 11 C, D) but started at 200 μM Mn^2+ ^(not shown). These data show that the reduction in ion fluxes in the mutant were indeed due to an alteration in entry mechanisms.

**Table 2:** Rate of basal and cAMP-induced Mn^2+^-influx. Amoebae were challenged with 1 or 100 μM Mn^2+ ^either with or without 1 μM cAMP. Cells were preincubated with 0.1 mM EGTA as outlined in Methods. Mn^2+ ^quenching of fura-2-dextran fluorescence was tested in H5-buffer and is expressed as decrease in fluorescence units/sec (mean ± s.e.m.). Numbers in brackets give number of cells tested and number of experiments.

Stimulation	Preincubation	
	none	EGTA (0.1 mM)
1 μM Mn^2+^	0 (121/5)	0.37 ± 0.2 (142/3)
1 μM Mn^2+^/1 μM cAMP	0 (135/5)	1.4 ± 0.1 (207/4)
		
100 μM Mn^2+^	0 (109/3)	
100 μM Mn^2+^/1 μM cAMP	1.0 ± 0.2 (292/9)	

In principle, the *iplA *gene product could form a channel in the plasma membrane or in membranes of internal stores. The lack of the *iplA *gene product in the stores might impair their coupling to the plasma membrane. As we had observed capacitative Ca^2+^-entry in the mutant we asked whether manipulation of the filling state of the stores altered ion fluxes. First we tested the effect of emptying of stores on Mn^2+^-influx. When cells were preincubated with EGTA, the requirement for high doses of Mn^2+ ^to quench fluorescence was abrogated. Now capacitative and also agonist-activated Mn^2+^-influx occurred at concentrations of MnCl_2 _comparable to those used under control conditions in wild type, in the range of 1–2 μM (Fig. 11 E, F). This result renders the possibility that the plasma membrane is altered in the mutant unlikely. Yet, the rate of Mn^2+^-influx observed in EGTA-treated mutant amoebae was still less than in wild type cells with respect to both basal and cAMP-activated fluxes (53 and 58% of wild type [12], respectively).

### [Ca^2+^]_i_-determination

**Figure 12:**
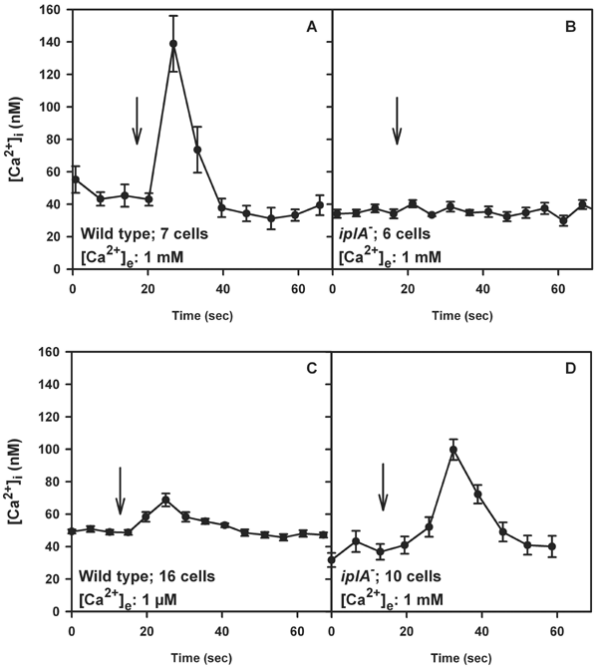
[Ca^2+^]_i_-recordings in cells preincubated with CaCl_2 _in order to load stores. (A) The response of wild type upon stimulation with 1 μM cAMP (arrow) at standard conditions, i.e. after preincubation with 1 mM CaCl_2 _for 10–15 min and stimulated in the presence of 1 mM CaCl_2 _is shown. Values give mean ± s.e.m. of 7 cells. (B) When *iplA*^- ^cells were stimulated with 1 μM cAMP at standard conditions (as outlined in (A)), no response was observed. Mean ± s.e.m. of 6 cells is shown. (C) Wild type was preincubated with 1 mM CaCl_2 _for 4–5 h; after washing cells were incubated in H5-buffer supplemented with 1 μM CaCl_2 _and challenged with 1 μM cAMP (arrow). Values give mean ± s.e.m. of 16 cells. (D) *iplA*^- ^incubated for 3 h with 20 mM CaCl_2 _were washed and subsequently [Ca^2+^]_i_-imaging was done in buffer containing 1 mM CaCl_2_. Arrow indicates the time point when 1 μM cAMP was added. Mean ± s.e.m. of 10 cells is shown.

In wild type cells treatment with EGTA augments responsiveness and cAMP-elicited [Ca^2+^]_i_-transients are detected at low extracellular [Ca^2+^] [12]. However, an agonist-induced [Ca^2+^]_i _elevation was not observed in *iplA*^- ^cells under these conditions. On the other hand, when stores were loaded in the continued presence of CaCl_2_, we observed that differentiation of the mutant resembled the wild type as described above. This led us to compare the [Ca^2+^]_i_-response of wild type and mutant amoebae after pretreatment with CaCl_2_. In wild type cells preincubation with 1 mM CaCl_2 _for 10–15 min and its continued presence during the [Ca^2+^]_i_-imaging experiment is required to activate a [Ca^2+^]_i_-transient after challenge with cAMP (Fig. 12 A and see [12]); in nominally Ca^2+^-free medium or at very low extracellular [Ca^2+^] a cAMP-activated [Ca^2+^]_i_-elevation is not observed [12]. In accordance with the data of Traynor et al. [6] this condition (Fig. 12 B) and even increasing the concentration of CaCl_2 _to 20 mM resulted in no detectable [Ca^2+^]_i_-increase in the mutant strain (not shown). However, we found that the basal [Ca^2+^]_i _level in *iplA*^- ^amoebae was significantly lower than in wild type and amounted to an average of 36 ± 3 nM (mean ± s.e.m. of 8 determinations in 3 independent experiments) as compared to 50 ± 2 nM in wild type (mean ± s.e.m. of 29 determinations in 9 independent experiments; Mann-Whitney rank sum test, p = 0.002).

For stronger loading of Ca^2+^-stores, we preincubated cells with 1 mM CaCl_2 _for 4 h. After this treatment a [Ca^2+^]_i_-elevation upon addition of cAMP was detected in 60% of wild type amoebae even at low extracellular [Ca^2+^] levels, i.e. when the buffer used to wash the cells had been supplemented with only 1 μM CaCl_2 _(Fig. 12 C). Starting from a basal level of 48 ± 4 nM, the height of the [Ca^2+^]_i_-transient amounted to 23 ± 2 nM (mean ± s.e.m. of 12 determinations in 8 independent experiments). Again, these conditions were not effective in *iplA*^- ^cells; rather, the sensitivity of the mutant was shifted to higher Ca^2+ ^concentrations as had been found with Mn^2+^-quenching experiments. We preincubated *iplA*^- ^amoebae with 20 mM CaCl_2 _for 3 h; after washing thoroughly, a cAMP-induced [Ca^2+^]_i_-elevation in the presence of 1 mM CaCl_2 _was observed (Fig. 12 D) in 28% of the cells; its average height amounted to 67 ± 11 nM (mean ± s.e.m. of 15 determinations in 4 independent experiments) starting from a basal level of 39 ± 2 nM. In the course of these experiments we once observed a response also under standard conditions, i.e. at 1 mM [Ca^2+^]_e _without prior incubation in 20 mM CaCl_2_; the height of the increase amounted to 54 ± 6 nM (mean ± s.e.m.). Yet, this was a rare event (once in 31 determinations).

## Discussion

The role of cAMP-activated [Ca^2+^]_i_-changes for chemotaxis has been questioned by Traynor et al. [6] who reported results obtained with the *iplA*^- ^mutant cell line favouring insignificance of [Ca^2+^]_i _for the chemotactic response. The authors had observed formation of fruiting bodies even though neither ^45^Ca^2+^-fluxes nor an agonist induced [Ca^2+^]_i_-elevation were detectable. The discrepancy between our view that a [Ca^2+^]_i_-elevation is necessary for a proper chemotactic response [5] and the conclusion of Traynor et al. prompted us to analyze chemotaxis, differentiation and the [Ca^2+^]-regulation of the *iplA*^- ^mutant in detail. In particular, we tested not only basal and cAMP-activated ion fluxes but also capacitative Ca^2+^-entry which is induced by emptying internal Ca^2+^-stores via preincubation of amoebae with EGTA [12].

Aggregation and development of wild type and mutant cells on agar plates was sensitive towards continuous emptying or loading of Ca^2+^-stores. These effects are not necessarily caused by altering chemotactic migration. It is conceivable that other Ca^2+^-dependent processes were affected, e.g. that the timing or pattern of gene expression or the establishment of cell contacts was altered. Although incubation of mutant amoebae for 2 h with 20 mM CaCl_2 _or of wild type with 1 mM CaCl_2 _for 4–5 h or with 1 mM EGTA for 1 h [12] did not significantly increase or lower basal [Ca^2+^]_i _it is possible that the continued presence of 10 mM EGTA or CaCl_2 _for many hours affects basal levels of [Ca^2+^]_i _which in turn might mediate effects on gene expression as was shown for prolonged incubation of cells with BHQ [18]. During development *Dictyostelium *cells form Ca^2+^-dependent and EDTA/EGTA-sensitive cell-cell contacts that are mediated by gp24 and DdCAD-1 ([19,20]; for review see [21]). Therefore, chelation of extracellular Ca^2+ ^might also inhibit cell adhesion. However, mutant cells whose gene encoding DdCAD-1 had been disrupted show normal chemotaxis and cell streams. Furthermore, mound formation was accelerated and only culmination was delayed by about 6 hours [20]. If only Ca^2+^-dependent cell adhesion was affected in our development assay in the presence of EGTA we would expect a similar phenotype. However, aggregation was clearly delayed. This argues for additional Ca^2+^-dependent processes during aggregation.

When we tested the influence of the presence of EGTA or CaCl_2 _on spontaneous motility and chemotaxis we found that in both strains motility in general was strongly impaired and that chemotaxis of the amoebae towards the cAMP-filled glass capillary was virtually abolished upon depletion of internal stores by the extracellular presence of EGTA. This effect is time dependent; after 30 min of incubation with 10 mM EGTA the behaviour of wild type amoebae was found to be unaltered and only after treatment for 0.5–1 h rounding and reduction of pseudopod elongation towards the capillary tip occurred [5]. Prolonged incubation for more than 1 h as carried out in this study completely inhibited the chemotactic response. These results strengthen the view that Ca^2+ ^has a necessary role in chemotaxis in wild type and in the mutant as well. When the cellular Ca^2+ ^content falls below a critical value Ca^2+^-dependent cytoskeletal rearrangements [22,23] that are necessary for both, random pseudopod extension during spontaneous motility and oriented pseudopod formation after chemotactic stimulation no longer take place correctly. On the other hand, the presence of 10 mM CaCl_2 _induced no alteration of basal cell motility in wild type or mutant amoebae. Yet, during chemotactic stimulation the average speed of migration towards the capillary tip was higher in mutant than in wild type cells. In this respect it is of importance that the basal level of [Ca^2+^]_i _was significantly lower in the former. At standard conditions the reduced basal [Ca^2+^]_i _does not impair the capacity of the mutant to chemotax. Therefore, this particular mutant strain represents the "minimal solution" with respect to the concentration of cytosolic Ca^2+ ^necessary to accomplish cytoskeletal rearrangements and extrusion of a pseudopod correctly. However, in the presence of 10 mM extracellular Ca^2+ ^during cAMP-stimulation Ca^2+^-fluxes are enhanced allowing more efficient formation of pseudopods. We had shown previously that a small global elevation of [Ca^2+^]_i _activates the extension of pseudopods all over the cell's circumference whereas a larger increase induces contraction of the amoebae [24]. In our view, the strongest evidence that a [Ca^2+^]_i_-transient is necessary for the extension of pseudopods rests upon the experiment where a Ca^2+^-chelator was introduced into the cytosol of the amoebae. This treatment led to rounding of the amoebae and a general reduction of pseudopod formation (see also [5]). Upon stimulation with a cAMP-filled capillary, the extension of oriented pseudopods was greatly reduced and migration towards the capillary tip was abolished. As Speksnijder et al. [11] had pointed out the fact that the presence of a chelator has an effect shows that a [Ca^2+^]_i_-gradient is essential for a given response. In summary, these data support the notion that an elevation of [Ca^2+^]_i _is required to extend pseudopods; suppression of the [Ca^2+^]_i_-elevation inhibits motility in general. Upon chemotactic challenge with cAMP this [Ca^2+^]_i_-gradient has to be established in a locally restricted fashion in order to allow local, oriented pseudopod formation (see [2,25,26]); otherwise pseudopods would be extended in all directions (see above, [24]). Our results imply that in *iplA*^- ^cells such a [Ca^2+^]_i_-gradient occurs as well, either nonrestricted allowing extension of pseudopods at random sites during spontaneous motility or restricted locally after chemotactic stimulation leading to oriented pseudopod formation. The fact that in the mutant cell line cAMP-activated [Ca^2+^]_i_-changes were practically undetectable under our standard condition argue for a [Ca^2+^]_i_-increase that is either smaller and/or more restricted to distinct domains within the cell than in wild type amoebae. Indeed, in only one out of roughly 30 determinations did we observe a cAMP-activated [Ca^2+^]_i_-transient under standard conditions. These results imply a crucial role but not an absolute necessity of the *iplA *gene product for the regulation of cAMP-induced [Ca^2+^]_i_-changes.

By using a Ca^2+^-sensitive electrode in cell suspensions, we analyzed which aspects of [Ca^2+^]_i _are controlled by the *iplA *gene product. Besides studying agonist-induced Ca^2+^-fluxes we also investigated capacitative Ca^2+^-entry and found that this type of influx was similar in mutant and wild type cell suspensions. We obtained equivalent results by testing Mn^2+^-quenching of Fura2-dextran fluorescence which showed that capacitative entry is independent of the *iplA *gene product.

On the other hand, using the Ca^2+^-sensitive electrode, we found that in the *iplA*^- ^mutant the agonist cAMP and also AA did activate Ca^2+^-entry into intact cells. The difference between the data published by Traynor et al. [6] and our results is most likely due to the experimental conditions: the magnitude of the Ca^2+^-fluxes that we observed was considerably lower than in wild type cells and detectable at low extracellular [Ca^2+^] only. The ^45^Ca^2+^-flux studies had been performed at 100 μM external CaCl_2_; so the fraction of ^45^Ca^2+ ^entering the cells was presumably too low to be detected reliably. Moreover, we found cAMP- and AA-induced Ca^2+^-release from stores in cells with permeabilized plasma membranes. These data show that cAMP-induced Ca^2+^-release from stores in *iplA*^- ^cells is functional. However, much like the Ca^2+^-influx, agonist-activated liberation from stores was smaller than in wild type amoebae. In line with these results are the findings using Mn^2+^-quenching to assay ion fluxes in intact single cells: higher doses of Mn^2+ ^were necessary to detect influx.

There are several interpretations for the results above. (i) There are two types of channels responsible for Ca^2+^-influx: one type being activated by emptying of the stores and sustaining capacitative Ca^2+^-entry which is unaffected in *iplA*^- ^cells and the other one mediating agonist-induced Ca^2+^-fluxes, the latter being under the control of the *iplA *gene product. The view that there are two strictly separated ion channels seems unlikely as under conditions of emptied stores cAMP-activated Mn^2+^-quenching occurred in the mutant as well. (ii) The same channel(s) mediate capacitative and agonist-activated fluxes but upon stimulation with agonists it cannot be addressed properly when *iplA *is disrupted. This implies a role of the protein in the liberation of Ca^2+ ^from the stores which is a prerequisite for the triggering of Ca^2+^-entry [12]. In the mutant this cannot proceed normally so subsequent activation of Ca^2+^-influx and the generation of a full [Ca^2+^]_i_-increase is impaired. The results of the experiments where stores were strongly loaded with Ca^2+ ^prior to stimulation support this notion. In this situation release from stores should be augmented. Indeed, in both, wild type and mutant cells, cAMP-activated [Ca^2+^]_i_-elevations occurred at an extracellular [Ca^2+^] (see Fig. 12) where without pretreatment no increase was observed. Presumably, release of Ca^2+ ^from the filled stores contributed to the observed [Ca^2+^]_i_-increase to a greater extent than under standard conditions. The requirement for 20 fold higher concentrations of CaCl_2 _during preincubation to elicit an agonist-induced [Ca^2+^]_i_-elevation in *iplA*^- ^cells are most likely due to the reduction in Ca^2+^-entry which necessitates a higher concentration gradient across the plasma membrane to fill the stores efficiently.

An as yet unresolved issue is the mechanism that induces Ca^2+^-entry upon liberation of Ca^2+ ^from the stores. From our data we conclude that in *Dictyostelium *these signals are different when the stores are emptied by EGTA or by agonist-activated signaling cascades. Otherwise one cannot explain normal capacitative Ca^2+^- and Mn^2+^-influx induced by EGTA-treatment and a requirement for 100 fold higher ion concentrations to induce Mn^2+^-entry by cAMP. If indeed the *iplA *gene product constitutes an IP_3_-receptor like channel that is located on membranes of stores the physical coupling of the receptor to channels in the plasma membrane as a mechanism to activate extracellular Ca^2+^-entry [27] should be missing in the mutant. On the other hand, emptying of stores by EGTA-treatment influences not only the IP_3_-sensitive store but also other stores and thus exerts a much more general effect on the cells. Studies using microarrays should reveal whether the expression of other genes is affected by the absence of *iplA *and thus might give a clue how [Ca^2+^]_i _is regulated in the mutant although one type of Ca^2+^-store is malfunctional.

## Conclusion

Our results show that Ca^2+ ^fluxes and regulation of Ca^2+ ^homeostasis take place in the *iplA*^- ^mutant and that chemotaxis and development of the mutant are sensitive to disturbance of the Ca^2+ ^homeostasis. In wild type cells and in cells lacking the *iplA *gene changes in [Ca^2+^]_i _are necessary to orient and to migrate chemotactically; their abolition causes loss of chemotaxis towards a cAMP source. The *iplA *gene product exerts a crucial role in the control of basal [Ca^2+^]_i _and of agonist induced Ca^2+^-fluxes. It is not required to activate capacitative Ca^2+^-influx. Thus the mechanisms responsible for capacitative and agonist-activated Ca^2+^-fluxes are different.

## Methods

### Materials

Fura2-dextran and Fura2 were purchased from MobiTec; cAMP was from Boehringer.

### Cell culture

*D. discoideum *wild type strain Ax2 and the *iplA*^- ^cell lines HM1049 and HM1038 (kindly provided by Dr. D. Traynor) were cultured as described [14] in the absence or presence of 10 μg/ml Blasticidin S, respectively. There was no difference between the two mutant strains with respect to the assays performed; therefore, results of measurements with either HM1038 or HM1049 are shown. Cells were washed by repeated centrifugation and resuspension in cold Sørensen phosphate buffer (17 mM Na^+^/K^+^-phosphate, pH 6.0). Amoebae were shaken at 2 × 10^7 ^cells/ml, 150 rpm and 23°C until use. The time, in hours, after induction of development is designated t_x_.

### [Ca^2+^]_e_-electrode recordings

[Ca^2+^]_e _in cell suspensions was recorded as described elsewhere [14]. Cells at t_5_–t_8 _were washed by repeated centrifugation and resupended at 5 × 10^7 ^cells/ml in 5 mM Tricine, 5 mM KCl, pH 7.0. Permeabilization was done by addition of filipin (15 μg/ml) to cell suspensions exactly as outlined in [15]. Capacitative Ca^2+^-influx was analyzed in cells with emptied storage compartments [12]: amoebae at t_2_–t_4 _were incubated with 5 mM EGTA for 30 min before washing in the above buffer.

### [Ca^2+^]_i_-determination and Mn^2+^-quenching experiments

Cells were loaded with Fura2-dextran (5 mg/ml + 1 mM CaCl_2_) at t_4_–t_5 _as described [12]. Aliquots (2–5 μl) of washed cells in H5-buffer (5 mM Hepes, 5 mM KCl, pH 7.0) were placed on glass coverslips and incubated in a humid chamber. 10–15 min prior to the experiment, 85–88 μl of H5-buffer + 1 mM CaCl_2 _were added. In a series of experiments to load stores, wild type and *iplA*^- ^cells were incubated with 1 mM CaCl_2 _for 4–5 h and with 20 mM CaCl_2 _for 2–3 h, respectively. Then they were thoroughly washed exactly as described previously [12] and incubated either in H5-buffer supplemented with 1 μM CaCl_2 _(wild type; free [Ca^2+^] in the solution was measured to be 2–2.5 μM, see also [12]) or in H5-buffer +1 mM CaCl_2 _(*iplA*^-^); final volume was 90 μl. Single cell [Ca^2+^]_i_-imaging was performed at t_7_–t_8 _as described [14]; stimulation was done by adding 10 μl of cAMP (10 μM). For Mn^2+^-quenching assays, washed cells were incubated in H5-buffer and challenged with Mn^2+ ^or Mn^2+^/cAMP. In order to study fluxes in cells with partially emptied internal storage compartments cells were preincubated with EGTA (10 μl of H5-buffer plus 0.1 mM EGTA for 1–2 h). 10–15 min prior to the experiment this solution was carefully removed and 100 μl of H5-buffer was added. This was repeated three times; final volume was 90 μl. Fluorescence quenching was measured at 360 nm excitation; influx rates are given as decrease of fluorescence units/sec.

### Measurement of Ca^2+^-fluxes in partially purified storage compartments

Analysis of vesicular Ca^2+^-fluxes was done as described [8]. In brief, 3 ml of cells at t_1_–t_6 _(2 × 10^8 ^cells/ml) in 20 mM Hepes, pH 7.2, were lysed by passage through Nuclepore filters. A final concentration of 3 % sucrose, 50 mM KCl, 1 mM MgCl_2_, 20 μg/ml leupeptin, 1 μg/μl aprotinin, 2.5 mM dithiothreitol and 1 μM microcystin were added; unbroken cells were removed by centrifugation at 3000 g for 5 min. The supernatant was centrifuged again at 12000 g for 20 min. The sediment (P) was resuspended in 1 ml of the above buffer. The rate of uptake and release was determined in the pellet and supernatant fraction by measuring the extravesicular [Ca^2+^] with Fura2.

### Chemotaxis assays

Cells were analyzed for chemotaxis towards a capillary filled with 0.1 mM cAMP [28]. 250 μl of 1 × 10^5 ^cells/ml in H5-buffer were pipetted onto a glass coverslip and allowed to settle for 60 min. Chemotaxis was recorded on a video recorder for 30–45 min. Chemotaxis was also assayed in the presence of EGTA or CaCl_2_; then amoebae were incubated in the respective agents for 60 min before they were challenged with cAMP. Images were digitized and the behaviour of the cells was analyzed using a computer program written for this purpose. For determination of cell velocity, a square area of interest (AI) of variable size (usually roughly 1/3 of the area of the cell) was placed at the perimeter of the cell in the first image digitized at the beginning of the assay. In the next image (images were digitized at a 2–4 sec time interval) the program analyzed an area larger than the AI (this area was defined by adding a given number of pixels on each side of the AI) for a pattern that resembled that of the AI; when such a pattern was found then the AI was placed on this new spot. The difference between the position of the AI in the first image to that in the second image was expressed as a vector of a given length. The changes in cell shape during migration were compensated by updating the pattern within the AI for every consecutive image analyzed. Calibration of the system allowed to convert the sum of the vector lengths to the distance in μm that the cells had migrated at the end of the experiment and to calculate the velocity of the amoebae. To test the effect of the intracellular presence of a Ca^2+^-buffer on chemotaxis, cells were loaded with Fura2-dextran (5 mg/ml in the loading solution) by electroporation in the absence of added external Ca^2+^. The amount of indicator present in the cytosol is in the range of 2–5% of the concentration present during electroporation [29]. 20 min after loading, cells were stimulated for 3–4 min by placing the cAMP-filled glass capillary at a distance of 10–20 μm of the cells and the number of cells that extended oriented pseudopods and thus elongated towards the capillary tip within this time period was counted. We had shown previously that loading of amoebae with FITC-dextran as a control does not alter chemotaxis as compared to untreated cells [5].

### Analysis of differentiation

Time lapse recordings of the development of Ax2 and *iplA*^- ^cells on 1.5 % agar in H5-buffer (H5-agar) were done by placing 4 × 10^6 ^cells each on one half of a petri dish (∅ 35 mm) at t_1_. The two populations were separated from each other by a thin plastic disc that had been inserted in the melted agar during cooling. Only after removal of fluid and slight drying of the plate the disc was removed which resulted in a thin rim separating the strains. Differentiation was recorded by capturing an image of the plate every 30 min using a stereo microscope (Stemi 2000, Zeiss) equipped with a CCD camera (AVT Horn) under the control of the AxioVision software package (Zeiss). In addition, development was assessed at various levels of extracellular CaCl_2_. Then H5-agar contained either 5–20 mM EGTA or 5–20 mM CaCl_2_.

## List of abbreviations

Cytosolic free Ca^2+ ^concentration: [Ca^2+^]_i_

2,5-di-(t-butyl)-1,4-hydroquinone: BHQ

Arachidonic acid: AA

Area of interest: AI

## Authors' contributions

RS recorded extracellular [Ca^2+^] in cell suspensions and participated in the design of the study. DFL participated in the recordings of extracellular [Ca^2+^] and the design of the study. KBR analyzed chemotaxis and differentiation of wild type and mutant cells. KH performed [Ca^2+^]_i _measurements. DM analyzed fluxes in partially purified storage compartments and was involved in the design of the study. CS participated in [Ca^2+^]_i _measurements, did Mn^2+^-flux studies, participated in the design of the study and wrote the manuscript.

## Supplementary Material

Additional File 1 Spontaneous cell motility of wild type and *iplA*^- ^cells in H5-buffer. Images of cells at t_5 _were captured every 15 sec for 20 min. Click here for file

Additional File 2 Basal cell motility after preincubation of wild type and mutant amoebae with 10 mM EGTA for 60 min (t_4_–t_5_). Images of cells at t_5 _were captured every 15 sec for 20 min in the continued presence of EGTA. Cells were rounded and extended smaller pseudopods than under control conditions. Click here for file

Additional File 3 Cell motility of wild type and mutant cells after preincubation with 10 mM CaCl_2 _for 80 min (t_4_–t_5.3_) is shown. Images of cells at t_5.3 _were captured every 15 sec for 20 min in the continued presence of CaCl_2_. The behaviour of treated cells was not different from control amoebae. Click here for file
